# Investigating the relationships among oral health-related knowledge, attitude, practice, and self-efficacy in predicting oral health behaviors among female Iranian students

**DOI:** 10.3389/froh.2025.1533519

**Published:** 2025-06-12

**Authors:** Akram Mehtari Taheri, Alireza Hidarnia, Fatemeh Zarei, Mahmoud Tavousi

**Affiliations:** ^1^ Faculty of Medical Sciences, Tarbiat Modares University, Tehran, Iran; ^2^ Health Metrics Research Center, Iranian Institute for Health Sciences Research, ACECR, Tehran, Iran

**Keywords:** oral health, knowledge, attitude, practice, self-efficacy

## Abstract

**Background:**

Dental caries and other oral health conditions, such as periodontal disease and halitosis, are significant global health issues, particularly among adolescents. Understanding how oral health-related knowledge, attitudes, practices, and self-efficacy contribute to the adoption of preventive oral health behaviors is therefore crucial. This study aimed to investigate the relationships among these factors in predicting oral health behaviors among female Iranian students.

**Methods:**

This cross-sectional analytical study was conducted from April to May 2023 and included 290 seventh-grade adolescent female students from schools in Kashan city. Data were collected via a validated questionnaire measuring knowledge, attitudes, practices, and self-efficacy regarding oral and dental health behaviors. Descriptive statistics, Pearson correlation, and regression analyses were performed via SPSS 24 software.

**Findings:**

The mean ± SD scores for knowledge, attitudes, self-efficacy, and practices were 5.72 ± 1.90, 56.22 ± 6.43, 49.81 ± 12.89, and 26.90 ± 6.28, respectively. Spearman's correlation coefficient revealed a strong relationship between self-efficacy and oral health behaviors (*r* = 0.657). Regression analysis revealed that self-efficacy was the most significant predictor of oral health behaviors (*β* = 0.349).

**Conclusion:**

The findings indicate that self-efficacy plays a key role in predicting oral health behaviors among adolescent students. Given its strong influence, educational interventions should focus on enhancing self-efficacy alongside improving knowledge, attitudes, and practices to promote better oral health behaviors.

## Background

Dental caries and other oral diseases are among the most prevalent global health issues, imposing significant health and economic burdens. These conditions severely impact individuals’ quality of life by causing pain, difficulty eating, and speech impairments (1–3). The Global Burden of Disease (2022) estimates that approximately 3.5 billion people worldwide suffer from oral diseases (4). Unlike many other infectious diseases, dental caries cannot be treated with antibiotics and can rapidly affect multiple teeth if left untreated (5). The World Health Organization (WHO) has classified dental caries as a major global health problem, with a prevalence of 60%–90% among school-aged children (6, 7).

In Iran, adolescents aged 12–14 are particularly vulnerable to dental caries, with a reported prevalence of approximately 60% and a mean Decayed, Missing, and Filled Teeth (DMFT) score of 1.84 (8, 9). A national screening survey conducted in 2014 revealed that first-year female high school students in some regions, such as Kashan, had the highest prevalence of dental caries, at 62% (10). Another study in Ahvaz, a major province of Iran, also indicated that dental caries was more common among adolescent girls than boys (11). Additionally, collaborative research by the WHO and the Iranian Ministry of Health reported a DMFT index of 67.1% (12), with higher rates among girls than boys (12, 13). The higher prevalence in females may be attributed to physiological, behavioral, and social factors, including dietary patterns and oral hygiene practices. However, further research is needed to better understand the sex differences in oral health.

Adolescence represents a critical period for establishing lifelong oral health habits. Several behavioral and environmental factors contribute to the high prevalence of dental caries among teenagers. Inappropriate nutritional choices, such as excessive sugar consumption and inadequate dairy intake, along with poor oral hygiene practices, including infrequent tooth brushing and a lack of flossing, contribute to an increased risk of dental caries (14, 15). The growing autonomy of adolescents often results in behavioral shifts, including changes in diet, smoking initiation, and the neglect of routine oral hygiene measures, which may lead to long-term oral health issues (16). Poor oral hygiene is associated with academic challenges, as the WHO estimates that more than 66 million school hours are lost annually due to oral health related problems (17, 18).In this study, oral health behavior was defined as regular engagement in preventive dental care activities, including bushing at least twice daily, daily flossing, and regular dental check-ups. These behaviors are essential for preventing dental caries and other oral diseases, particularly during adolescence, when habits are formed (19).

## Self-Efficacy and oral health behaviors

Self-efficacy, a core concept in Bandura's social cognitive theory, plays a crucial role in shaping health-related behaviors, including oral health practices (20). It refers to an individual's belief in their ability to successfully perform a behavior. Bandura identifies four key sources of self-efficacy: (1) Mastery experiences—successfully performing a behavior increases confidence in one's ability to repeat it; (2) Vicarious experiences—observing others engaging in a behavior can enhance self-efficacy; (3) Verbal persuasion—encouragement from peers, parents, or educators strengthens self-efficacy; and (4) Physiological and emotional states—emotional well-being can influence confidence in performing behaviors (21, 22). By clearly understanding and reinforcing these sources, adolescents may be more likely to adopt and maintain positive oral health behaviors. Scheerman et al. (23) reported that self-efficacy was a significant predictor of toothbrushing frequency among adolescents aged 12–17 years (23). Similarly, Dolatabadi et al. (24) reported a strong association between oral health self-efficacy and adherence to preventive dental care among teenagers (24). A systematic review by Scheerman et al. (25). identified self-efficacy as one of the key psychosocial predictors of oral health behaviors, emphasizing its importance in adolescent populations (25). Despite the well-established knowledge‒attitude‒practice (KAP) model in oral health research, its limitation in accounting for self-efficacy as a behavioral determinant remains largely overlooked, particularly among adolescent girls (26). Given the greater prevalence of dental caries in this group, understanding the role of self-efficacy in shaping oral health behaviors is essential. While existing studies have focused primarily on knowledge and attitudes, few have explored how self-efficacy interacts with these factors to influence behavioral outcomes. This study addresses this gap by (1) specifically examining female students, who are at greater risk for dental issues in certain regions; (2) utilizing a validated instrument to assess psychological and behavioral determinants of oral health; and (3) providing empirical evidence on the predictive role of self-efficacy in behavior change. By doing so, this study aims to enhance the theoretical understanding of adolescent oral health behaviors and inform the development of school-based interventions that foster both self-efficacy and preventive practices. Ultimately, this research seeks to investigate the interplay between knowledge, attitudes, practices, and self-efficacy in predicting oral health behaviors among adolescent girls.

## Methods

### Study design, setting, and location

This cross-sectional analytical study was conducted from April to May 2023 with 290 female seventh-grade students in Kashan, Iran. The main purpose of this study was to investigate the relationships among knowledge, attitudes, practices, and self-efficacy in predicting oral health behaviors in Iranian female school students.

### Participant recruitment and inclusion/exclusion criteria

The study's inclusion criteria specified that participants must be female seventh-grade students currently enrolled in school who also provided informed consent to be involved in the research. A national oral health screening survey conducted in 2014 reported that seventh grade female students in regions such as Kashan had the highest prevalence of dental caries (62%) compared with other age groups. Given this alarming prevalence, targeting this specific population allowed for a more focused investigation into the factors influencing oral health behaviors at a critical developmental stage (10). The exclusion criteria included students who had either dropped out of school, emigrated from Kashan city, or were unable to perform dental hygiene practices due to physical limitations.

### Sampling and sample size

A systematic cluster sampling method was used. The 37 schools in Kashan were divided into five clusters (north, south, east, west, and center), and two schools were randomly selected from each cluster. A total of 10 schools were selected, and one seventh-grade class was randomly selected from each school. The sample size was calculated via Cochran's formula, considering a 0.05 margin of error and a 95% confidence level, resulting in a sample size of 240. Considering a 20% dropout rate in cluster sampling, 290 students were ultimately included in the study. Kashan has 37 urban middle schools with 6,828 students.

$$n=\frac{\frac{Z^{2}pq}{d^{2}}}{1+\frac{1}{N}\left(\frac{Z^{2}pq}{d^{2}}-1\right)}n=\frac{\frac{3.8\times 0.2\times 0.8}{0.0025}}{1+\frac{1}{6828\left(\frac{3.8\times 0.2\times 0.8}{0.0025}-1\right)}}≅240$$

n: Statistical sample size

N: the size of the statistical population d: Error (0.05) z: for the 95% confidence level is equal to 1.96

p: the ratio of possessing the desired attribute (0.2) (the number of changes in the desired attribute)

q: Proportion of individuals not having the desired trait (0.08)

### Data collection instrument

The questionnaire used in this study was selected because of its validated structure and strong psychometric properties, which have been previously tested in adolescent populations (27). It assesses multiple dimensions of oral health, including knowledge, attitudes, practices, and self-efficacy, which are crucial determinants of behavior change. The self-efficacy section of the questionnaire was specifically designed to measure self-efficacy related to maintaining oral health behaviors such as tooth brushing, flossing, and attending dental check-ups. It does not assess general self-efficacy, but rather domain-specific confidence in performing preventive oral health actions. The content validity index (CVI) and content validity ratio (CVR) for this instrument were above 0.62 and 0.79, respectively, indicating strong reliability and validity. Moreover, given the cognitive and developmental stages of adolescents, a self-administered questionnaire is the most practical and effective method for assessing their perceptions and behaviors. The questionnaire comprises two parts:

#### Demographics

Questions on age, parents' education level, parents' occupation, number of family members, and dental visits.

#### Constructs

Knowledge (12 items): Each correct answer is given a score of 1, with a total score ranging from 0 to 12. Higher scores indicate greater knowledge.

• Attitude (13 items): Rated on a 5-point Likert scale from “strongly agree” (5) to “strongly disagree” (1), with a total score ranging from 13 to 65. Higher scores indicate a better attitude.
• Self-efficacy (14 items): Confidence in brushing and flossing was assessed on a 5 point Likert scale from “completely confident” (5) to “not at all confident” (1), with a total score ranging from 14 to 70.
• Practice (8 items): Rated on a 5-point Likert scale (always, most of the time, sometimes, rarely, never), with a total score ranging from 8 to 40. Higher scores indicate better oral health practices. The content validity index (CVI) and content validity ratio (CVR) are greater than 0.62 and greater than 0.79, respectively. With respect to the reliability of the questionnaire, the alpha coefficient was above 0.7 (27). (The questionnaire is available in the Supplementary File.)

### Data collection process

After cluster sampling and identifying the list of schools, one seventh-grade class was selected from each school. The researcher coordinated with the school principals and visited the schools on specific days to distribute the questionnaires to the students. The questionnaires were completed by the students.

### Outcome, predictor, and confounder variables

The outcome variable was oral health behaviors. The predictive variables in this study are knowledge, attitudes, and self-efficacy in brushing and flossing. Confounding factors, such as parents' education level, parents' occupation, number of family members, and number of dental visits, were controlled for in the analysis to ensure that the observed associations were not due to these factors.

### Ethical considerations

The study adhered to the Declaration of Helsinki and received ethical approval from the Human Ethics Committee at the University of Tarbiat Modares, Tehran, Iran (IR.MODARES.REC.1402.052). Written informed consent was obtained from all parents and/or legal guardians for their children's participation in the study, and they were assured that in all stages of data collection, data entry, and preparation of the final report, their personal information would be kept confidential and that the release of data would be performed as a group.

### Data analysis

The normality of the collected data was assessed via the Kolmogorov‒Smirnov test in SPSS version 24, which indicated that the data did not follow a normal distribution. Therefore, nonparametric statistical tests were applied to ensure the validity of the findings. Descriptive statistics, including measures of central tendency and dispersion for quantitative variables, as well as frequency and percentage for qualitative variables, were computed. To examine the relationships between the study variables, the Kruskal‒Wallis, Mann‒Whitney U, and correlation tests were employed. Additionally, regression analysis was conducted to identify predictors of oral health behaviors. A *p* value of less than 0.05 was considered statistically significant.

## Findings

### Demographic characteristics

The present study was conducted on 290 seventh-grade students in Kashan, Iran. The participants' average age was 12.8 ± 0.57 years, ranging from 12 to 14 years. The minimum and maximum number of family members were 3 and 8, respectively, with an average family size of 4.41 ± 0.89. According to the findings, 36.9% of the students' fathers had middle school educations, 42% had diplomas and associate educations, and 15.5% had bachelor's and higher educations. Additionally, 35.9% of the students' mothers had middle school education, 41.1% had diploma and associate education, and 19% had bachelor's and higher education. The majority of fathers (43.1%) were self-employed, and most mothers (76.9%) were housewives. Most students lived in their own homes (73.1%) and had visited a dentist (75.2%) (Table 1).

**Table 1:** Demographic characteristics of adolescent girls students.

Variables	Subgroup	Frequency	Percentage
Father's education	Middle school	107	36.9
	Diploma and associate degree	122	42
	Bachelor's degree and above	45	15.5
Mother's education	Middle school	103	35.6
	Diploma and associate degree	119	41.1
	Bachelor's degree and above	55	19
Father's occupation	worker	77	26.6
	Employee	58	20.0
	Self- employed	125	43.1
Mother's occupation	Housewife	223	76.9
	Employee	25	8.6
Rank of children	First	137	47.2
	Second	108	37.2
	Third and above	45	6.4
Housing status	Own house	212	73.1
	Rental house	69	23.8
Dental visits	Yes	218	75.2
	No	67	23.1

### Relationships between oral health behaviors and demographic characteristics

The results of the Kruskal‒Wallis and Mann‒Whitney *U*-tests revealed no significant relationships between the mean score of oral health behaviors and fathers' education (*p* = 0.48), fathers' occupation (*p* = 0.84), or mothers' occupation (*p* = 0.45). However, there was a significant relationship between the mean score of oral health behaviors and mothers' education (*p* = 0.03). Additionally, there was a significant relationship between the knowledge construct and the father's education (*p* = 0.02), the mother's education (*p* = 0.007), and the number of dental visits (*p* = 0.02). In other words, the mean scores of oral health behaviors, including knowledge and dental visits, were significantly higher in students whose parents had higher education levels (Table 2).

**Table 2:** Comparison of the mean scores of preventive oral health behavior with demographic factors in female student.

Significance level variables		Father's education	Mother's education	Father's occupation	Mother's occupation	Number of family members	Dental visits
Knowledge	*p*-value	0.02	0.007	0.33	0.87	0.26	0.02
Attitude	*p*-value	0.62	0.19	0.55	0.77	0.63	0.25
Self-efficacy	*p*-value	0.43	0.47	0.98	0.28	0.97	0.71
Practice	*p*-value	0.48	0.03	0.84	0.45	0.97	0.09

### Comparing the mean scores of the oral health behaviors and self-efficacy constructs

The mean (SD) scores for knowledge, attitudes, self-efficacy, and practices related to oral health behaviors were 5.72 ± 1.90, 56.22 ± 6.43, 49.81 ± 12.89, and 26.90 ± 6.28, respectively. The results revealed that among the three domains of preventive oral health behaviors, the highest score was related to the attitude construct, with a mean score of 56.22 ± 6.43, and the lowest score was related to the knowledge construct, with a score of 5.72 ± 1.90. Among the four dimensions of self-efficacy in brushing and flossing, verbal encouragement had the highest mean score of 8.07 ± 2.6, and the lowest score in the field of self-efficacy was related to physical and emotional status, with a score of 8.69 ± 19.64 (Table 3).

**Table 3:** Comparison of the mean scores of preventive oral health behavior domains and self-efficacy domains of adolescent girls students.

Domains	Subgroups	Values (Mean ± SD)	Percentage of score obtained	Minimum and maximum achieved score
Preventive Oral Health Behaviors	Knowledge	5.72 ± 1.90	47.5	0–12
	Attitude	56.22 ± 6.43	86.4	13–65
	Practice	26.90 ± 6.28	67.2	40–8
Self-efficacy	Mastery experience	13.86 ± 5.12	69	4–20
	Observational experience	7.88 ± 2.51	78	2–10
	Verbal encouragement	8.07 ± 2.6	80	2–10
	Physical & emotional state	19.64 ± 8.69	65.3	6–30
	All areas of self-efficacy	49.81 ± 12.89	71.1	14–70

### Correlations between constructs

According to the results, there was a strong, direct, and linear correlation between oral health behaviors and the self-efficacy construct (*r* = 0.657) and a moderate and direct correlation between oral health behaviors and the attitude construct (*r* = 0.461). Additionally, a moderate and direct correlation was observed between self-efficacy in oral health and the attitude construct (*r* = 0.541). However, a weak, direct, and linear correlation was observed between knowledge about oral health and the self-efficacy construct (*r* = 0.304) and between knowledge about oral health and the attitude construct (*r* = 0.242). These relationships were statistically significant (*p* < 0.05) (Table 4).

**Table 4:** Correlation matrix of oral health behaviors and knowledge, attitude, and self-efficacy constructs of adolescent girls students.

Variable	Knowledge	Attitude	Self-efficacy	Behavior
Knowledge	1	–	–	–
Attitude	0.304**	1	–	–
Self-efficacy	0.242**	0.541**	1	–
Behavior	0.202*	0.461**	0.657**	1

* *p* value less than 0.05.

** *p* value less than 0.001.

### Regression test analysis

Table 5 shows the regression test results for the prediction of oral health behaviors on the basis of the knowledge, attitudes, and self-efficacy constructs. The self-efficacy construct, with a coefficient of B (0.349), was the most important predictor of oral health behaviors. With a one-unit increase in the self-efficacy construct score, the mean score of oral health behaviors significantly increased (0.34) units. The construct of self-efficacy in brushing and flossing predicted 57% of the dependent variable, oral and dental health behaviors.

**Table 5:** Regression table of predictors of oral health behaviors in adolescent girls students.

Variables	Beta (β)	Lower bound	Upper bound	*P*-value
Constant	5.261	1.731	11.159	0.005
Self -efficacy	0.349	0.320	0.375	0.003
Attitude	0.093	−0.006	0.147	0.075
Knowledge	−0.095	−0.368	0.300	0.056

*R*^2^ = 0.57.

## Discussion

This study was conducted to investigate the multiple relationships among knowledge, attitudes, practices, and self-efficacy in predicting oral health behaviors among female school students.

### Factors influencing oral health behaviors

The results of this study revealed that there was no significant relationship between the mean score of oral health behaviors and parents' occupation. However, a significant relationship was observed between the mean score of oral health behaviors and the mother's education. In the studies of Gudarzi et al. (8) and Elamin et al. (28), the low education level of mothers was significantly related to tooth decay. Additionally, a significant relationship was found between the knowledge construct and parents' education and number of dental visits (8). These findings suggest that parents' education level, particularly mothers', plays a role in their children's oral health behaviors. This could be explained by the fact that mothers are typically more involved in their children's daily lives and healthcare, including oral health (11, 29). The study revealed that the students lacked adequate knowledge about oral health. Only 47% of them were aware of oral health preventive measures, and only 23% and 32% of the students were aware of the importance of flossing and mouthwash, respectively. Taheri et al. (30) and Marashi et al. (31) reported that the awareness and knowledge of people in the field of oral health were not sufficient. This lack of knowledge could be attributed to various factors, including the lack of comprehensive oral health education programs in schools, the influence of cultural beliefs and practices, and the limited access to oral health information and resources.

The study revealed that the students had a positive attitude toward oral health behaviors, with more than 80% of them expressing a favorable attitude. Similar findings were reported in other studies (32, 33). This positive attitude could be attributed to the importance that individuals, especially adolescents, place on physical appearance and their desire to maintain healthy and beautiful teeth. However, Lawal et al. (34) reported poor attitudes among students about oral health, which may be attributed to living in low-income areas with limited access to education and health facilities.

However, it seems that this positive attitude does not always translate into appropriate behavior. The construct of self-efficacy should be given special attention since it can have a strong relationship with the occurrence of behavior and is a prelude to performing a behavior. Therefore, self-efficacy and increased self-confidence are necessary to perform oral and dental hygiene behaviors during adolescence. In this study, the self-efficacy of the students was 71%, and in the studies of Mueller et al. (33) and Dolatabadi et al. (24) In the study of Bantel et al. (35) High self-efficacy in teenage students in the oral and dental fields is associated with an increase in general health, quality of life, and oral and dental hygiene and an increase in brushing frequency and a decrease in fear and anxiety. Woelber et al. (36) noted low self-efficacy among their target groups and acknowledged that individuals with low self-efficacy were more likely to experience gingival bleeding problems.

The study emphasized the importance of focusing on various aspects of self-efficacy, including physical and emotional state, mastery experience, observational experience, and verbal encouragement. By providing adequate information and raising awareness about the importance of brushing, flossing, and visiting a dentist, students can develop a high level of self-efficacy through regular practice (37). Importantly, self-efficacy in oral health behavior should be increased both quantitatively (frequency of brushing and flossing) and qualitatively (correct technique of brushing and flossing). Role modeling is also an important strategy for improving self-efficacy (20). In this vein, students who perform well in oral health can be identified and introduced as role models for other students. Additionally, many behaviors are learned by adolescents through observation and imitation by their parents (37). Therefore, parents should be thoroughly educated about oral health practices so that they can participate more actively in their children's oral health education process. Adolescents may not be able to brush and floss regularly due to a lack of tools to overcome temptation, peer pressure, or lack of motivation. Under these circumstances, additional strategies are needed to help them maintain these routines consistently. This role can be fulfilled by teachers and parents, who can provide encouragement and verbal motivation to the students. Therefore, self-efficacy, as a strong predictor, can encourage individuals to engage in healthy behaviors (31). As shown in Table 3, the percentage of the total score obtained for oral health behaviors was 67.2%, indicating moderate adherence to recommended oral health practices among the participants. However, the mean scores for brushing after each meal, flossing at least once a day, and visiting the dentist were 37%, 32%, and 18%, respectively. In the study of Opoku et al. (38), there was a significant relationship between students' knowledge and level of education and compliance with oral health. Costa-Pazos et al. (39) discussed the importance of self-esteem in adolescents and noted that those with high self-esteem tend to perform well in oral hygiene behaviors. These findings highlight the need for effective interventions to improve oral health behaviors among adolescents (40, 41).

The results of the correlation test revealed a strong, direct correlation between oral health behaviors and the self-efficacy construct, indicating that self-efficacy is a strong predictor of behavior change (33, 42). This finding is consistent with those of previous studies. This relationship can be explained by individuals with higher self-efficacy setting higher goals for themselves, expecting better outcomes, perceiving barriers and challenges as surmountable, and being more likely to engage in self-care behaviors. A moderate, direct correlation was also observed between oral health behaviors and the attitude construct and between self efficacy in oral health and the attitude construct (32). These findings suggest that attitudes play a role in shaping oral health behaviors but are not the only factor involved. Self-efficacy, which is more closely related to action, appears to be a stronger predictor of behavior.

### Predictors of oral health behaviors

The findings of the present study provide evidence of the importance and predictive effect of self-efficacy on oral health behaviors. This model revealed that 57% of the changes related to oral health behaviors are explained by self-efficacy. Woelber et al. (36) and Haerian Ardakani et al. (43) reported that brushing and flossing self-efficacy is a good predictor of students' oral and dental health. Additionally, health officials and practitioners can design intervention programs on the basis of the findings of this study.

### Limitations and future research

One limitation of this study was the self-reported nature of the data collected through questionnaires. Future studies should utilize more objective indicators and information, such as decayed, missing, and filled teeth (DMFT) scores. Another limitation was the noncooperation of some school principals. To address this limitation, future studies should involve collaboration with relevant educational authorities. Additionally, this study was conducted at Kashan, where students had relatively good oral health. Future studies should be conducted in rural areas to investigate the status of this behavior and its related constructs in this population group. Furthermore, future studies should focus on longitudinal studies and educational interventions in the field of oral health self-care behaviors for all population groups on the basis of behavioral models.

## Conclusion

On the basis of the findings of this study, to improve oral health behaviors, it is necessary to consider individual, environmental, and lifestyle factors related to oral health behaviors and health policies in this field. According to the study results, self-efficacy is a key determinant of self-care behaviors in the oral health of adolescent students. Therefore, incorporating educational materials on oral self-care into the school curriculum can be beneficial. It is recommended to increase adolescents' awareness and improve their health.

## Data Availability

The original contributions presented in the study are included in the articles/supplementary material, further inquiries can be directed to the corresponding author.
