# Supplementary material for: Investigating the relationships among oral health-related knowledge, attitude, practice, and self-efficacy in predicting oral health behaviors among female Iranian students
Source: Front Oral Health. 2025 Jun 12;6:1533519. doi: 10.3389/froh.2025.1533519 (PMC12198220; doi:10.3389/froh.2025.1533519)
Supplement: Supplementary file 1 [file Datasheet1.docx]

**Supplementary file :**

**Assessment of knowledge, attitudes, practices, and self-efficacy regarding oral and dental health.**

**Questionnaire ID: ............. Completion Date: ........**

Dear Student,

This questionnaire is part of a research project on oral and dental health to assess knowledge, attitudes, practices, and self-efficacy regarding oral and dental health. All collected information will be analyzed collectively and used to improve students’ health. Please note that the information in this questionnaire is strictly confidential and will only be accessed by the researchers. Thank you for your cooperation in advance.

**Knowledge Questions (Please choose only one option for each statement):**

1. Which of the following foods contributes most to tooth decay?

a. Eggs and meat

b. Fruits and vegetables

c. Sugary foods such as chocolates

d. I don’t know

**2. Which of the following statements is correct about brushing frequency?**

a. After every meal

b. At least twice a day

c. Both a and b

d. I don’t know

**3. How much toothpaste should be applied to the toothbrush?**

a. Pea-sized amount

b. Full length of the toothbrush

c. Half the length of the toothbrush

d. I don’t know

**4. What is the best way to clean between teeth?**

a. Toothbrush

b. Dental floss

c. Mouthwash

d. I don’t know

**5. How often should a toothbrush be replaced?**

a. Every month

b. Every three months

c. Every six months

d. I don’t know

**6. Which of the following statements is correct about using dental floss?**

a. After every brushing

b. Before every brushing

c. Once a day

d. I don’t know

**7. What effect does fluoride-containing mouthwash have on teeth?**

a. Helps teeth grow

b. Whitens teeth

c. Reduces tooth decay

d. I don’t know

**8. How long should you avoid eating or drinking after using fluoride mouthwash?**

a. 30 minutes

b. 1 hour

c. 2 hours

d. I don’t know

**9. Which of the following is correct about using fluoride mouthwash?**

a. After brushing

b. Before brushing

c. Once a week

d. I don’t know

**10. Which of the following shows the correct order for brushing teeth?**

a. Upper jaw first, then lower jaw

b. Lower jaw first, then upper jaw

c. I don’t know

**11. What is the appropriate duration for brushing teeth?**

a. 30 seconds

b. At least 1 to 4 minutes

c. More than 5 minutes

d. I don’t know

**12. How often should you visit a dentist for a check-up?**

a. Every 6 months

b. Once a year

c. Only when experiencing tooth pain

d. I never visit a dentist

Attitude

| **Statement** | **Strongly Agree** | **Agree** | **Neutral** | **Disagree** | **Strongly Disagree** |
| --- | --- | --- | --- | --- | --- |
| 1. Eating sweets, chocolates, and sugar causes tooth decay. | ☐ | ☐ | ☐ | ☐ | ☐ |
| 2. I believe drinking milk is very important for dental health. | ☐ | ☐ | ☐ | ☐ | ☐ |
| 3. I believe if I don’t brush my teeth, they will decay faster. | ☐ | ☐ | ☐ | ☐ | ☐ |
| 4. I believe brushing alone doesn’t clean all parts of the teeth, and dental floss is necessary for cleaning between teeth. | ☐ | ☐ | ☐ | ☐ | ☐ |
| 5. I believe one reason for bad breath is not regularly using a toothbrush and dental floss. | ☐ | ☐ | ☐ | ☐ | ☐ |
| 6. I believe using mouthwash along with a toothbrush and dental floss is essential for dental health. | ☐ | ☐ | ☐ | ☐ | ☐ |
| 7. I like to have beautiful and healthy teeth. | ☐ | ☐ | ☐ | ☐ | ☐ |
| 8. I must develop the habit of brushing my teeth after meals. | ☐ | ☐ | ☐ | ☐ | ☐ |
| 9. I must use dental floss at least once a day. | ☐ | ☐ | ☐ | ☐ | ☐ |
| 10. I believe that in addition to using a toothbrush and dental floss, I should use mouthwash at least once a week. | ☐ | ☐ | ☐ | ☐ | ☐ |
| 11. I believe if my mouth smells bad, others won’t like talking to me. | ☐ | ☐ | ☐ | ☐ | ☐ |
| 12. Even if my friends don’t clean their teeth, I will brush mine. | ☐ | ☐ | ☐ | ☐ | ☐ |
| 13. Even if my parents don’t care about cleaning their teeth, I will brush mine. | ☐ | ☐ | ☐ | ☐ | ☐ |

Self-efficacy

| **Statement** | **Completely Confident** | **Confident** | **Moderately Confident** | **Slightly Confident** | **Not Confident at All** |
| --- | --- | --- | --- | --- | --- |
| 1. If cleaning my teeth is challenging, I am confident I can clean them. | ☐ | ☐ | ☐ | ☐ | ☐ |
| 2. I am confident I can brush my teeth three times a day, even if I am busy. | ☐ | ☐ | ☐ | ☐ | ☐ |
| 3. I am confident I can brush my teeth after every snack. | ☐ | ☐ | ☐ | ☐ | ☐ |
| 4. Despite the time-consuming nature of flossing, I am confident I can floss every night. | ☐ | ☐ | ☐ | ☐ | ☐ |
| 5. Seeing my family members brush their teeth makes me confident I can brush mine. | ☐ | ☐ | ☐ | ☐ | ☐ |
| 6. Watching videos of toothbrushing and flossing makes me confident I can brush and floss my teeth. | ☐ | ☐ | ☐ | ☐ | ☐ |
| 7. I am confident my parents’ compliments about my clean teeth will encourage me to brush my teeth. | ☐ | ☐ | ☐ | ☐ | ☐ |
| 8. I am confident my classmates' and teachers’ encouragement about my clean teeth will motivate me to brush. | ☐ | ☐ | ☐ | ☐ | ☐ |
| 9. Even if I am very tired, I am confident I can clean my teeth. | ☐ | ☐ | ☐ | ☐ | ☐ |
| 10. Despite having a lot of homework, I am confident I can clean my teeth. | ☐ | ☐ | ☐ | ☐ | ☐ |
| 11. Even during vacations (traveling or visiting guests), I am confident I can clean my teeth. | ☐ | ☐ | ☐ | ☐ | ☐ |
| 12. Even if I am watching TV or playing, I am confident I can clean my teeth. | ☐ | ☐ | ☐ | ☐ | ☐ |
| 13. I am confident I can brush and floss my teeth even when I feel bored. | ☐ | ☐ | ☐ | ☐ | ☐ |
| 14. Even if I am sick, I am confident I can brush and floss my teeth. | ☐ | ☐ | ☐ | ☐ | ☐ |

Practice

| **Statement** | **Always** | **Most of the Time** | **Sometimes** | **Rarely** | **Never** |
| --- | --- | --- | --- | --- | --- |
| 1. I brush my teeth after eating sweets and snacks. | ☐ | ☐ | ☐ | ☐ | ☐ |
| 2. I ask my parents to take me to the dentist every 6 months, even if I don't have a toothache. | ☐ | ☐ | ☐ | ☐ | ☐ |
| 3. In the past week, I have flossed my teeth at least once a day. | ☐ | ☐ | ☐ | ☐ | ☐ |
| 4. In the past week, I have rinsed my teeth with mouthwash at least once. | ☐ | ☐ | ☐ | ☐ | ☐ |
| 5. In the past week, I have brushed my teeth after every meal. | ☐ | ☐ | ☐ | ☐ | ☐ |
| 6. In the past week, I have eaten fruits during break time or as an afternoon snack. | ☐ | ☐ | ☐ | ☐ | ☐ |
| 7. In the past week, I have consumed snacks such as puffed corn, cake, or chips. | ☐ | ☐ | ☐ | ☐ | ☐ |
| 8. In the past week, I have consumed milk and dairy products at least once a day. | ☐ | ☐ | ☐ | ☐ | ☐ |

**Demographic characteristics**

**Dear Student,**
Please read the questions in this section and answer questions 1 to 4 in the blanks provided. For the remaining questions, mark the appropriate box with a cross (X).

**Background and Personal Information:**

1. Age: ..................... years
2. First Name: ...................... Last Name: ........................... School Name: .......................
3. Number of family members: ..................... persons
4. Weight: .......... kg Height: .......... cm
5. What is your birth order in the family?
   - 1. First child
     2. Second child
     3. Third child
     4. Fourth child
     5. Fifth child
     6. Sixth child or later
6. Have you ever visited a dentist for filling or extraction of your teeth?
   - 1. Yes
     2. No
7. Father’s education:
   - 1. Illiterate
     2. Primary school
     3. Middle school
     4. High school diploma
     5. Associate degree
     6. Bachelor’s degree
     7. Master’s degree or higher
8. Mother’s education:
   - 1. Illiterate
     2. Primary school
     3. Middle school
     4. High school diploma
     5. Associate degree
     6. Bachelor’s degree
     7. Master’s degree or higher
9. Father’s occupation:
   - 1. Worker
     2. Employee
     3. Unemployed
     4. Self-employed
     5. Other
     6. Please specify: .........................
10. Mother’s occupation:
    1. Housewife
    2. Employee
    3. Other

Please specify: .........................

1. What type of house do you live in?
   1. Owned house
   2. Rented house
   3. Other

Please specify: .........................
